# A rebuttal to the comments on the genome order index and the *Z*-curve

**DOI:** 10.1186/1745-6150-6-10

**Published:** 2011-02-16

**Authors:** Ren Zhang

**Affiliations:** 1 Department of Epidemiology and Biostatistics, Tianjin Cancer Institute and Hospital, Tianjin 300060, PR China

## Abstract

**Background:**

Elhaik, Graur and Josic recently commented on the genome order index (*S*) and the *Z*-curve (Elhaik et al. Biol Direct 2010, 5: 10). *S *is a quantity defined as *S *= *a*^2 ^+ *c*^2 ^+ *g*^2 ^+ *t*^2^, where *a*, *c*, *g *and *t *denote corresponding base frequencies. The *Z*-curve is a three dimensional curve that represents a DNA sequence in the manner that each can be uniquely reconstructed given the other. Elhaik et al. made 4 major claims. 1) In the previous mapping system with the regular tetrahedron, calculation of the radius of the inscribed sphere is "a mathematical error". 2) *S *follows an exponential distribution and is narrowly distributed with a range of (0.25 - 0.33). 3) Based on the Chargaff's second parity rule (PR2), "*S *is equivalent to *H *[Shannon entropy]" and they are derivable from each other. 4) *Z*-curve "suffers from over dimensionality", because based on the analysis of 235 bacterial genomes, *x *and *y *components contributed only less than 1% of the variance and therefore "would be of little use".

**Results:**

1) Elhaik et al. mistakenly neglected the parameter $4/\sqrt{3}$ when calculating the radius of the inscribed sphere. 2) The exponential distribution of *S *is a restatement of our previous conclusion, and the range of (0.25 - 0.33) only paraphrases the previously suggested *S *range (0.25 -1/3). 3) Elhaik et al. incorrectly disregard deviations from PR2 by treating the deviations as 0 altogether, reduce *S *and *H*, both having 4 variables, *a, c, g *and *t*, into functions of one single variable, *a *only, and apply this treatment to all DNA sequences as the basis of their "demonstration", which is therefore invalid. 4) Elhaik et al. confuse numeral smallness with biological insignificance, and disregard the distributions of purine/pyrimidine and amino/keto bases (*x *and *y *components), the variations of which, although can be less than that of GC content, contain rich information that is important and useful, such as in locating replication origins of bacterial and archaeal genomes, and in studies of gene recognition in various species.

**Conclusion:**

Elhaik et al. confuse *S *(a single number) with *Z*-curve (a series of 3D coordinates), which are distinct. To use *S *as a case study of *Z*-curve, by itself, is invalid. *S *and *H *are neither equivalent nor derivable from each other. The criticisms of Elhaik, Graur and Josic are wrong.

**Reviewers:**

This article was reviewed by Erik van Nimwegen.

## Background

The debate originated from a paper published in 1991, in which we defined a quantity *S *= *a*^2 ^+ *c*^2 ^+ *g*^2 ^+ *t*^2^, where *a*, *c*, *g *and *t *denote corresponding base frequencies in a DNA sequence, and we studied *S *values for protein coding genes [1]. In 2004, we calculated *S *values for genome sequences, and found that *S *< 1/3 is valid for most genomes [2]. In 2008, Elhaik et al. criticized this work with 2 claims [3]. 1. *S *<*S *1/3 is in fact a mathematical property that is always true regardless of specific data. 2. *S *and *H *(Shannon entropy) are strictly equivalent. To rebut with minimum space, I raised one counterexample to each claim [4]. 1. When *a *= *c *= 0.5, *g *= *t *= 0, *S *= 0.5, which is larger than 1/3; thus *S *< 1/3 is not a mathematical property that is always true, and it depends on specific data to be valid. 2. *H*, but not *S*, has the property of additivity of information entropy; they thus differ. Therefore, both claims are incorrect [4].

In a more recent comment published in *Biology Direct *[5], Elhaik et al. dropped the first claim, but still insisted on the equivalence of *S *and *H*. Furthermore, they made additional criticisms, which, however, are once again incorrect, and the reasons are summarized in this rebuttal. To make it easy to follow, subtitles here correspond to the ones in [5].

## Results and Discussion

### Inscribed sphere or circumscribed sphere?

In [1], we introduced a method that maps a DNA sequence onto a point within the regular tetrahedron (RT). In [2], we showed that for most genomes, *S *< 1/3, that is, the mapping points are within the inscribed sphere of the RT. Elhaik et al. claimed that "the inscribed sphere calculations were erroneous", and the conclusion that *S *< 1/3 and the mapping points are within the inscribed sphere is "a consequence of a mathematical error", because they noted that the radius of the inscribed sphere of the RT involved is 1/4 rather than $1/\sqrt{3}$[5]. Using 235 bacterial genomes, they found that the mapping points of 45% of these genomes were outside the inscribed sphere [5]. Their calculation is incorrect, due to the neglect of a parameter, as shown below.

Letting the nucleotide frequencies of A,C, G and T be denoted by *a*, *c*, *g *and *t*, respectively, and taking the center of the RT as the origin, a coordinate system (*X, Y, Z*) can be set up [1]

(1) $$\left\{ \begin{array}{l} X=\frac{\sqrt{3}}{4}\left[\left(a+g)-(c+t\right)\right],\\ Y=\frac{\sqrt{3}}{4}\left[\left(a+c)-(g+t\right)\right],\\ Z=\frac{\sqrt{3}}{4}\left[\left(a+t)-(g+c\right)\right],X,Y,Z\in [-\frac{\sqrt{3}}{4},\frac{\sqrt{3}}{4}], \end{array}\right.$$

where *X*, *Y *and *Z *are the coordinates of the mapping point P in this coordinate system. For convenience, we introduced a reduced coordinate system (*x*, *y*, *z*) such that (refer to the equation (3) of the reference [1])

(2) $$\left\{ \begin{array}{l} X=\frac{\sqrt{3}}{4}x,\\ Y=\frac{\sqrt{3}}{4}y,\\ Z=\frac{\sqrt{3}}{4}z. \end{array}\right.$$

Then we have

(3) $$\left\{ \begin{array}{l} x=(a+g)-(c+t),\\ y=(a+c)-(g+t),\\ z=(a+t)-(g+c),\;\;\;x,y,z\in [-1,1]. \end{array}\right.$$

Equation (3) is the one that has been mostly used in related studies. Based on equation (2),

(4) $$R=\frac{\sqrt{3}}{4}r,$$

where *R *and *r *are the radii of the inscribed sphere in the original (*X, Y, Z*) and the reduced (*x, y, z*) coordinate systems, respectively. Therefore, in the reduced coordinate system the radius of the inscribed sphere is

(5) $$r=\frac{4}{\sqrt{3}}R=\frac{\text{4}}{\sqrt{\text{3}}}\times \frac{\text{1}}{\text{4}}=\frac{1}{\sqrt{3}}.$$

One can use either the original (*X*, *Y*, *Z*) or the reduced coordinate system (*x, y, z*), but should use the corresponding radius, *R *(1/4) or ($1/\sqrt{3}$), for the former and latter, respectively. Elhaik et al. used the equation (3) to obtain the coordinates in the reduced coordinate system (*x, y, z*), but still used *R *(1/4), the radius in the original system (*X*, *Y*, *Z*) [5]. In other words, their mistake is due to the confusion of the original and reduced coordinate systems.

**Figure 1:**
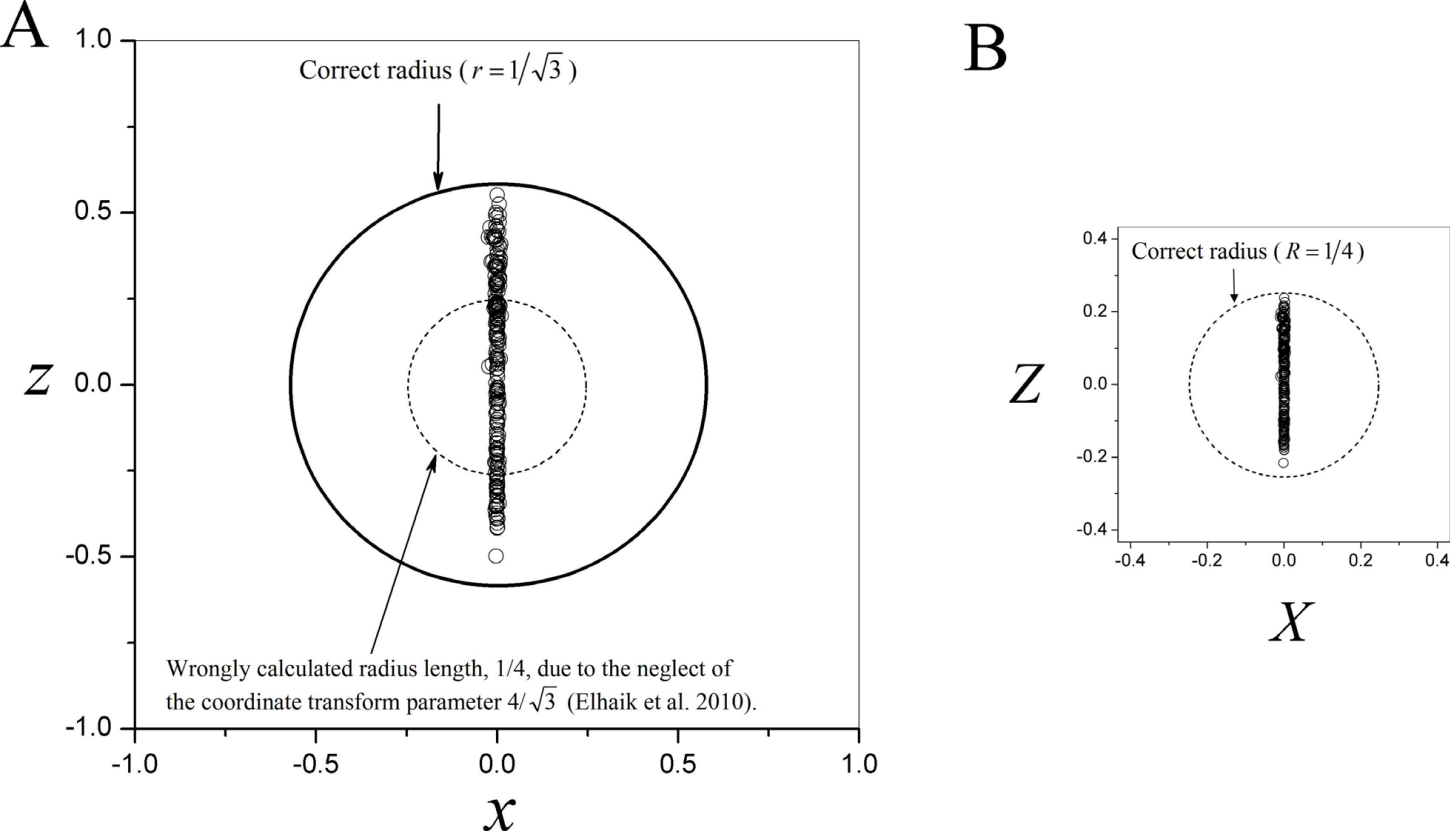
**The distribution of the mapping points for the 235 bacterial genomes used in **[5]. A) The distribution of the mapping points in the reduced coordinate system (*x, y, z*) according to equation (3). The square (the side length = 2) corresponds to the projection of the regular tetrahedron onto the *x*-*z *coordinate plane. The bold and dotted circles correspond to projections of inscribe spheres with the correct radius length $1/\sqrt{3}$ and wrongly calculated value 1/4, respectively. Based on the correct radius, in contrast to their conclusion that 45% of genomes have mapping points outside the inscribed sphere, none has the mapping point outside the inscribed sphere and none has an *S *value larger than 1/3. B) The distribution of the mapping points in the original coordinate system (*X, Y, Z*) according to equation (1). The square (the side length = $\sqrt{3}/2$) corresponds to the projection of the regular tetrahedron onto the *X-Z *coordinate plane. Note that all mapping points are within the inscribed sphere, too, whose radius length is 1/4. The mistake of Elhaik et al. is the confusion of the original and reduced coordinate systems, and consequently, the neglect of the coordinate transform parameter $4/\sqrt{3}$. Refer to text for details.

Figure 1 shows the distribution of the mapping points of the 235 bacterial genomes used in [5]. Based on the correct radius, in contrast to their conclusion that 45% of genomes have mapping points outside the inscribed sphere, none has the mapping point outside the inscribed sphere and none has an *S *value larger than 1/3. Therefore it is the inscribed sphere, as correctly indicated in the original article [2]. The mistake of Elhaik et al. is the confusion of the original and reduced coordinate systems, and consequently, the neglect of the parameter $4/\sqrt{3}$.

### *S *is narrowly distributed

In this section, Elhaik et al. first "found that the distribution of *S *values follows an exponential distribution". This is a restatement of our previous conclusion [2], albeit with less data. Compare the figure 2 in [2] and the figure 2 in [5]; in the former more than 800 genomes of multiple species were studied, while in the latter, only 235 bacterial genomes were used. Elhaik et al. then found that *S *is narrowly distributed with a range of (0.25 - 0.33), which only paraphrased our previously suggested *S *range (0.25 -1/3) [2].

### Is *S *equivalent to *H*?

In 2005 [6], we proposed a new algorithm for genome segmentation, and we indicated that the algorithms based on either *S *or *H *are equivalent in obtaining segmentation points for binary sequences composed of two nucleotide types, A/T and G/C bases. Note that here the word "equivalent" has a context, that is, in a specific application (genome segmentation for binary sequences) with a specific algorithm that we proposed, and it does not mean *S *and *H *are equivalent.

In their first comment [3], Elhaik et al. extrapolated this conclusion, without any proof, claiming that, "*S *is strictly equivalent to *H*", which is incorrect as one counterexample was enough to show they are not equivalent (Only *H *has the property of additivity of information entropy) [4].

In [5], they claimed to have demonstrated "*S *is equivalent to *H*", and "*S *is completely determined from the [Shannon] entropy". Their demonstration is under the assumption that in a DNA sequence, *a *= *t *and *g *= *c *(eq. A1), and *a *+ *c *= 0.5 (eq. A2), and consequently, *S *and *H*, both having 4 variables, *a*, *c*, *g *and *t*, were reduced into functions of one single variable, *a *only (eq. A3). This demonstration is invalid.

First, *S *is used to study any DNA sequences, not necessarily only whole genomes. For instance, to perform genome segmentation studies, *S *can be calculated for sequences shorter than 1 Kb [6]. In [1], *S *of protein coding genes was studied. For single genes or DNA sequence segments, not necessarily, base compositions of A and T (or C and G) are exactly the same, and the sum of A and C is exactly 50% (eq. A1 and A2). That is, the "=" signs in their eq. A1 and A2, the basis of the demonstration, are invalid. *S *and *H*, both being functions of all the 4 base frequencies, should not be reduced into functions of one single variable, the composition of the nucleotide A only (eq. A3 and A5, [5]). For instance, for the human *Sirtuin-3 *gene (AF083108), the *S *value is 0.265 (*a *= 0.174, *c *= 0.279, *g *= 0.333 and *t *= 0.214). However, according to their function (eq. A3) in [5], the *S *value becomes 0.273. Apparently 0.265 ≠ 0.273. In other words, *S *and *H *cannot be determined by the composition of one single nucleotide. Therefore, their demonstration is invalid.

For whole genome sequences, their demonstration is still incorrect. It is well known that Chargaff's second parity rule (PR2) [7] holds for most genomes (note that not all genomes obey PR2 [8]). It is instructive to know that according to PR2 in single-stranded DNA, *a *and *t *(or *c *and *g*) are equal approximately, but it can never be assumed that these base compositions are exactly the same. In fact, deviations of PR2, which result from both mutation and selection pressures, reflecting biases in, for instance, DNA replication, transcription and repair, have been an important subject of study in the past decades [9]. Therefore, the "=" signs in their equations A1 and A2 are still invalid. For instance, for the genome of *X. fastidiosa *9a5c (NC_002488), one of the 235 bacterial genomes that Elhaik et al. selected, *a *(22.54%) ≠ *t *(24.78%), *c *(24.94%) ≠ *g *(27.73%), and *a *+ *c *= 0.475 ≠ 0.50.

Elhaik et al. wrote "We note that the relation between *S *and *H *[...] may not hold for DNA sequences that violate the second parity rule, such as organellar DNA and single stranded DNA sequences. However, even these genomes obey a less stringent rule: that the number of *a *+ *g*'s approximately equals the number of *t *+ *c*'s". Here, two issues need clarification. First, not only in organellar DNA and single stranded DNA sequences, in other genomes, also, the compositions *a *+ *g *and *t *+ *c *are only equal approximately. Second, for those genomes that obey a more "stringent" PR2 rule, these compositions are still only equal approximately, but can never be assumed as exactly the same. Deviations from PR2, although small, contain critical and rich information, and these deviations differ among genomes, and they thus should not be simply disregarded and treated as 0 altogether, i.e., for genomes, *S *and *H *still should not be treated as functions of the composition of one single nucleotide A only (eq. A3 and A5 in [5]). Therefore, for genomes their demonstration is still invalid.

In some cases, e.g., for whole genomes, *S *and *H *can be correlated. In 2004, we first indicated that *S *and *H *are negatively correlated for genomes [2], hence the name genome order index. In fact, both *S *and *H *are special forms of the *α*-order entropy [10]. Suppose that there is a random variable *X *~ *p*(*x*), the *α*-order entropy is defined as

(6) $$\begin{array}{ccc} H^{\alpha }(X)=\frac{1}{1-\alpha }\left(\sum_{x\in X}{\left[p(x)\right]}^{\alpha }-1\right), & \alpha >0, & \alpha \neq 1. \end{array}$$

It was shown that

(7) $$\lim_{\alpha \to 1}H^{\alpha }(X)=H(X)=-\sum p(x)\log p(x),$$

where *H *(*X*) is the Shannon entropy. Specially, if *α *= 2, eq. (6) reads

(8) $$H^{2}(X)=\left(1-\sum_{x\in X}{\left[p(x)\right]}^{2}\right)=1-S=1-\sum_{n=1}^{4}p_{n}^{2}$$

where the last two equal signs are valid only for a discrete uniform distribution of four alphabetic symbols. As I indicated in [4], *S *is a linear transformation of a special case (4 alphabetic symbols) of *H*^2 ^(*X*), the Gini-Simpson index. This index is a general information index used in many areas, while *S*, which is derived from a totally independent way, is a special one for the analysis of DNA sequences, and additionally, *S *has a clear geometrical meaning, i.e., *S *is proportional to the square of the distance between a point and the RT origin in the mapping system that we proposed [1].

Generally, *S *and *H *are neither equivalent nor derivable from each other. For whole genomes, they can be correlated. The relation between *S *and *H *is complex; as shown in [11], the relation differs in different cases, e.g., different value ranges. In genome segmentation studies, *S *outperforms *H *by having a faster computation time, which is especially important for handling large genomes, such as the human genome [6].

### Does Z-curve have over dimensionality?

In this section, Elhaik et al. switch topic from *S *to *Z*-curve, claiming that *Z*-curve suffers from "over dimensionality". This conclusion is based on the analysis of 235 bacterial genomes. Using principal component analysis (PCA), Elhaik et al. found that "99.91% of the variance is accounted for by the *z *coordinate, and the *x *and *y *coordinates accounted for 0.053% and 0.003% of the variance, respectively". They conclude that "the *z *axis is, therefore, the only meaningful coordinate for studying nucleotide composition." and *x *and *y *contribute only less than 1% of the variance and therefore "would be of little use". Assuming that the 235 bacterial genomes that they selected were indeed representatives of all genomes, including those of eukaryotes, viruses and archaea, which is unlikely, the process reaching this conclusion is still logically flawed.

First, they confuse numeral smallness with biological insignificance. A biological process with a readout that is numerically small does not necessarily mean it is biologically unimportant. For genomes, according to PR2, *a *≈ *t *and *g *≈ *c*. Based on equation (3), without doing PCA, obviously, |*z*| >> |*x*| ≈ |*y*| ≈ 0. That is, *x *and *y *components are small numbers that are close to 0. However, it does not necessarily mean that *x *and *y *components, i.e., variations of purine/pyrimidine and amino/keto bases, respectively, along the genome, are not important. For instance, based on *x *and *y *components, replication origins have been located in more than 1000 bacterial genomes [12,13], and also in archaeal genomes [14]. For example, for archaea *Sulfolobus solfataricus *and *Aeropyrum pernix*, analysis based on *x *and *y *components predicted multiple replication origins [14,15], which are consistent with later experimental results [16,17].

Second, *Z*-curve can be used in analyzing any DNA sequences, such as protein coding genes [18], promoter sequences [19] and translation start sites (TSS) [20]. Protein coding genes or DNA sequence segments in various species do not necessarily have the same nucleotide variation patterns as the one in the 235 bacterial genomes, the basis of their conclusion. For instance, based on *Z*-curve behaviors, bacterial TSS can be reliably predicted, and for sequences around bacterial TSS, *x *and *y *components in fact have more variations than the *z *component, in contrast to the variation pattern of bacterial genomes [20]. *Z*-curve based algorithms have been successfully used in recognizing protein coding genes in genomes of budding yeast [18], bacteria and archaea [21], viruses and phages [22], especially coronaviruses [23] and in recognizing short coding sequences of human genes [24]. In all these algorithms, *x *and *y *components are absolutely needed to achieve high gene recognition accuracy.

In this section, the major mistake (among some others, such as incorrectly extrapolating a result based on a subset of bacterial genomes to those for all DNA sequences) of Elhaik et al. is the confusion of numeral smallness with biological insignificance. Variations of purine/pyrimidine and amino/keto bases (*x *and *y *components) should not be disregarded and treated as "little use" only because they could be small in magnitude; in contrast, they are important and useful. As mentioned above, based on *x *and *y *components, a large number of replication origins have been located in both bacterial [12,13] and archaeal genomes [14]. The *x *and *y *components play an absolutely indispensable role in *Z*-curve based gene finding algorithms, which have been successfully applied in recognizing protein coding genes in, to name a few, the genomes of *L. interrogans *Lai [25], *B. amyloliquefaciens *FZB42 [26], *B. thuringiensis *BMB171 [27], *A. mediterranei *U32 [28], *M. tuberculosis *H37Ra [29], *Drosophila *[30], new human coronaviruses HCoV-NL63 [31] and HKU1 [32], four coronaviruses from bats [33], new phages Rtp in *E. coli *[34] and in a pandemic *V. parahaemolyticus *O3:K6 strain [35].

## Conclusions

In many cases the statements by themselves [3,5] make little sense. Below are some examples.

1. "The genome order index was selected as a case study to the usefulness of the *Z*-curve method." *S *is a statistical quantity (one single number), while *Z*-curve is a 3-dimensional curve that constitutes a one-to-one correspondence of a DNA sequence (a series of 3-D coordinates). *S *is not *Z*-curve, and *S *cannot be used as a case study of *Z*-curve.

2. "We must conclude that both the *Z*-curve and *S *are over complicated measures to GC content and Shannon *H *index, respectively." *Z*-curve is not a measure of GC content. *S *is not a measure of Shannon *H *index. If *Z*-curve were a measure of GC content, it would be striking that gene recognition can be achieved with a high accuracy [18,21,22,24] based solely on GC content.

3. "the dimension stands for GC content alone suffices to represent any given genome." GC content alone does not suffice to represent any given genome, simply because the genome is composed of 4 kinds of nucleotides, and distributions of purine/pyrimidine and amino/keto bases should not be disregarded only because their variations can be less than that of the GC content.

4. Elhaik, Graur and Josic finally concluded that "the genome order index is a misconceived mathematical tool that should not be used in a meritorious sequence analyses." This conclusion is, by itself, not consistent. The Shannon entropy is a well-established method that has been widely used in many areas. Elhaik et al. on the one hand claim that *S *is strictly equivalent to the Shannon entropy, and on the other hand claim that *S *is a misconceived mathematical tool; then the next logical conclusion would be the Shannon entropy is a misconceived mathematical tool, which is obviously against scientific commonsense.

In summary, Elhaik, Graur and Josic (i) confuse the reduced coordinate system with the original one, and consequently, mistakenly neglected the parameter $4/\sqrt{3}$ when calculating the radius of the inscribed sphere. (ii) The exponential distribution of *S *is a restatement of our previous conclusion, and the range of (0.25 - 0.33) only paraphrases the previously suggested *S *range (0.25 -1/3). (iii) Elhaik et al. incorrectly disregard deviations from PR2 by treating the deviations as 0 altogether, reduce *S *and *H*, both having 4 variables, *a, c, g *and *t*, into functions of one single variable, *a *only, and apply this treatment to all DNA sequences as the basis of their "demonstration", which is therefore invalid. Importantly, they confuse numeral smallness with biological insignificance, and disregard the distributions of purine/pyrimidine and amino/keto bases, the variations of which, although sometimes less than that of GC content, contain rich information that is important and useful. Therefore, the criticisms of Elhaik, Graur and Josic are wrong.

## Materials and methods

The same 235 bacterial genomes (based on genome names) that were used by Elhaik et al. in [5] were analyzed. The data in Table S1 in ref. [5] contain numerous mistakes. The Table S1 contains 4 columns, genome name, size, GC content and ID. Eighteen IDs correspond to plasmids, not genomes. These IDs are: NC_007410, NC_006873, NC_004943, NC_003080, NC_007414, NC_007515, NC_007801, NC_007483, NC_007274, NC_007336, NC_007901, NC_007641, NC_006855, NC_007608, NC_005951, NC_006663, NC_005229 and NC_004554. Calculation of genome length and GC content is incorrect for many genomes. For instance, the calculated GC content for *B. fragilis *YCH46 (NC_006347) was 33.50% [5], while the correct number is 43.27%. The calculated GC content for *C. acetobutylicum *ATCC 824 (NC_003030) was 37.00% [5], while the correct number is 30.93%.

## Competing interests

The author declares that they have no competing interests.

## Authors' contributions

RZ analyzed the data and wrote the manuscript. The author read and approved the final manuscript.

## Reviewer comments

This manuscript, seems to be the latest shot in an ongoing dispute between this author and Elhaik et al. regarding the usefulness of certain statistics for analyzing base composition of DNA. After looking at this manuscript and the paper that it is a rebuttal to, I must say that I am amazed that so much debate can arise over issues that are essentially very basic (i.e. how to summarize base composition in one or a few statistics) and I am wondering how useful these kinds of exchanges are for general readers.

Much of the discussion centers around the DNA-sequence statistic S, which is defined as the sum of the square-frequencies of the letters: S = (f_a)^2, + (f_c)^2 + (f_g)^2 + (f_t)^2 where f_a, f_c, f_g, and f_t are the base frequencies. Clearly, since f_a + f_c + f_g + f_t = 1, we necessarily have that S lies in the range [0.25,1]. Both this author and Elhaik et al. seem to agree that, for a large collection of bacterial genomes, we find S < 1/3 but there is disagreement about how 'surprising' this is and what kind of constraint that this is indicative of. First of all, it is clear that for uniformly random sequences the frequencies f_x will be close to 0.25 and thus S will be close to 0.25 as wll. Only for extremely biased base compositions would one get values of S close to 1 and so, in my opinion, it is not 'surprising' at all that there that one does not find genomes with large S values. One might reasonably argue, in my opinion, that the surprising observation is that one gets S values as HIGH as 0.33.

A second point of contention is whether the S statistic and the entropy H = -sum_x f_x log(f_x) are 'equivalent'. The dispute here seems to mostly be of a semantic nature, i.e. regarding the meaning of the word 'equivalent'. I can only see two relevant points: 1) For large DNA sequences (like whole genomes) it is observed that there is an approximate symmetry between the two DNA strands, i.e. the base composition in one strand is not significantly different from the base composition in the other strand. Since, by Watson-Crick base-pairing rules, we only have C-G/G-C and A-T/T-A pairs, this implies that APPROXIMATELY f_a = f_t and f_c = f_g (*)

Now, if we assume that the equalities (*) hold exactly, then we have three constraints

f_a+f_c+f_g+f_t = 1

f_a = f_t

f_c = f_g

and so we effectively have only 1 degree of freedom left (which is essentially GC-content). Since both S and H are invertible functions of the remaining degree of freedom, it immediately follows that S can be calculated from H and H from S. Whether you want to call this equivalent or not is a matter of semantics. The point is that when all three constrains are acting, there is only one degree of freedom left. Instead of calculating S or H, I think it would be much more straight-forward to just talk about GC-content directly. Indeed, it is remarkable that CG-content ranges from as low as 0.22 to as high as 0.77 and the relevant biological question, in my opinion, is not whether to use S or H or whatever other derived statistic, but rather trying to explain why GC-content varies so much in bacterial genomes. Indeed there has been quite some interesting developments in this area recently. See for example the discussion in: Rocha EP, Feil EJ. Mutational patterns cannot explain genome composition: are there any neutral sites in the genomes of bacteria? PLoS Genet. 2010 Sep 9;6(9).

The discussion about Renyi entropies is useless in my opinion. Yes, both S and H are both members of a family of functions (Renyi entropies) but I fail to see how this is relevant for any biological question.

Of course, in reality one only has that f_a is approximately equal to f_t (and similar for f_c and f_g). Thus, H and S may vary independently. However, because the equalities almost always very nearly hold, and because H and S are smooth functions of the base frequencies, there is still a very tight quantitative relation between H and S in real data. Thus, I agree with Elhaik et al. that the variation of S and H across different genomes is dominated by the variation in GC-content.

2) The remaining question is whether there is any biological meaning in the deviations from f_c = f_g and f_a = f_t. The current author makes the valid point, in my opinion, that numerically small deviations may still be meaningful biologically. The author asserts in several places that, indeed, these deviations are highly meaningful but frustratingly fails to give citations to back this claim up. My own recollection is that in bacteria the G/C-skew has been proposed to be a result of different mutational spectra acting on the leading and lagging strands (and would thus not necessarily have functional implications).

The author does later cite a number of papers that use the Z-curve statistic to find genes and replication origins and states that the components orthogonal to GC-content are crucial for these methods. I immediately believe this to be correct. For example, as we and others have found the presence of ribosomal binding sites plus the avoidance of RNA secondary structure around the translation start site leads to clear base-compositional biases around the starts of genes (Eyre-Walker and Bulmer Nucl. Acids Res 1993, Molina & van Nimwegen Genome Res 2008). However, this seems to now confound the question of local compositional biases and their functional implications versus global patterns of base composition, because as far as I can tell Elhaik et al. were talking about global compositional patterns.

Finally, the remark that S can be calculated faster than H 'which is especially important for handling large genomes' does not make a lot of sense to me. If one really worries about computational costs in calculating H one could calculate f*log(f) for all values of before-hand and store them in a table.

### Author's response

Elhaik, Graur and Josic made 4 major claims, which are rebutted. The review report, although long, evades 2 major points being debated. The first 2 claims made by Elhaik et al. are: 1) The conclusion that the mapping points of most genomes are within the inscribed sphere, i.e., S < 1/3, is a consequence of mathematical error. 2) S follows an exponential distribution. I point out that their first claim is incorrect due to the neglect of a coordinate transform parameter and their second claim is only a restatement of our previous conclusion. Both points are not touched in the review report, and I therefore presume the reviewer has no objection to my rebuttal. The reviewer, however, does disagree with my rebuttal but agree with Elhaik et al. on some issues, to which I will respond point by point.

## Reviewer comments

I am amazed that so much debate can arise over issues that are essentially very basic

It is not 'surprising' at all that there that one does not find genomes with large S values

I am wondering how useful these kinds of exchanges are

The discussion about Renyi entropies is useless

### Author's response

I agree that some issues are basic. For instance, their first claim is due to mistakenly neglecting a parameter in coordinate transformation, which belongs to elementary mathematics.

However, first, here the issue is not about whether a topic is basic or not, surprising or not, useful or not; it is about right or wrong. Regarding the questions such as whether the calculation of the inscribed sphere radius is 'a mathematical error', and whether Z-curve suffers from 'over dimensionality', there is only one answer: yes or no. Science literatures and readers deserve the truth. Second, in contrast, whether a topic is surprising or useful is largely a personal opinion. Therefore I will not further discuss whether certain issues are basic/surprising/useful.

## Reviewer comments

A second point of contention is whether the S statistic and the entropy H = -sum_x f_x log(f_x) are 'equivalent'. The dispute here seems to mostly be of a semantic nature, i.e. regarding the meaning of the word 'equivalent'. I can only see two relevant points:

1) For large DNA sequences (like whole genomes) it is observed that there is an approximate symmetry between the two DNA strands, i.e. the base composition in one strand is not significantly different from the base composition in the other strand. Since, by Watson-Crick base-pairing rules, we only have C-G/G-C and A-T/T-A pairs, this implies that APPROXIMATELY f_a = f_t and f_c = f_g (*)

Now, if we assume that the equalities (*) hold exactly, then we have three constraints

f_a+f_c+f_g+f_t = 1

f_a = f_t

f_c = f_g

and so we effectively have only 1 degree of freedom left (which is essentially GC-content). Since both S and H are invertible functions of the remaining degree of freedom, it immediately follows that S can be calculated from H and H from S. Whether you want to call this equivalent or not is a matter of semantics. The point is that when all three constrains are acting, there is only one degree of freedom left. Instead of calculating S or H, I think it would be much more straight-forward to just talk about GC-content directly. Indeed, it is remarkable that CG-content ranges from as low as 0.22 to as high as 0.77 and the relevant biological question, in my opinion, is not whether to use S or H or whatever other derived statistic, but rather trying to explain why GC-content varies so much in bacterial genomes. Indeed there has been quite some interesting developments in this area recently. See for example the discussion in: Rocha EP, Feil EJ. Mutational patterns cannot explain genome composition: are there any neutral sites in the genomes of bacteria? PLoS Genet. 2010 Sep 9;6(9). The discussion about Renyi entropies is useless in my opinion. Yes, both S and H are both members of a family of functions (Renyi entropies) but I fail to see how this is relevant for any biological question.

### Author's response

Throughout the criticisms and the rebuttal, when debating on S and H, the only Chargaff Parity Rule being referred to is the parity rule 2 (PR2). Note that PR2 is a phenomenon in one single DNA strand (a ~ = t and c~ = g), but not double DNA strands. Indeed, in a duplex DNA, a = t and c = g, due to Watson-Crick base pairing, but that is the Chargaff Parity Rule 1.

The reviewer's discussion is based on the phenomenon in 2 DNA strands. The reviewer writes: "symmetry between the two DNA strands, i.e. the base composition in one strand is not significantly different from the base composition in the other strand... Since by Watson-Crick base-pairing rules, we only have C-G/G-C and A-T/T-A pairs ...". The debate is about PR2, a phenomenon of base compositions in the DNA single strand, while the reviewer's discussion is about DNA double strands. Because of this misunderstanding, the reviewer's discussion about S and H becomes almost irrelevant.

## Reviewer comments

Thus, I agree with Elhaik et al. that the variation of S and H across different genomes is dominated by the variation in GC-content.

### Author's response

Here the reviewer agrees with Elhaik et al. for a point that Elhaik et al. did not intend to make. Elhaik et al. studied the variations of Z-curve's 3 components (x,y,z) using 235 bacterial genomes, and found that the z component (which is related to GC content) contributed to most of the variance, comparing to x and y components (please refer to the figure 4 in ref. [5]). Note that the studied variations are about Z-curve, not related to S and H.

Nevertheless, it is true that distributions of S and H are indeed quite related to the GC content. But that is a conclusion made by myself in the original article. Please refer to the figure 3 in ref. [2] and the text therein.

## Reviewer comments

The author asserts in several places that, indeed, these deviations are highly meaningful but frustratingly fails to give citations to back this claim up. My own recollection is that in bacteria the G/C-skew has been proposed to be a result of different mutational spectra acting on the leading and lagging strands (and would thus not necessarily have functional implications).

### Author's response

Deviations from PR2 result from both mutation and selection pressures, reflecting biases in, e.g., DNA replication, transcription and repair. I added a review article.

## Reviewer comments

However, this seems to now confound the question of local compositional biases and their functional implications versus global patterns of base composition, because as far as I can tell Elhaik et al. were talking about global compositional patterns.

### Author's response

No. Elhaik et al. concluded that Z-curve suffers from "over-dimensionality", without restricting their conclusion to global compositional patterns only. Z-curve can be used to study any DNA sequences, such as whole genomes, protein coding genes, promoter sequences and translation start sites. Therefore, one part of their analysis that is logically flawed is that they analyzed a subset of bacterial genomes but tried to make a general conclusion for all DNA sequences. In my rebuttal, however, I have to show separately that for both whole genomes and short DNA segments, their conclusion is wrong.

## Reviewer comments

Finally, the remark that S can be calculated faster than H 'which is especially important for handling large genomes' does not make a lot of sense to me. If one really worries about computational costs in calculating H one could calculate f*log(f) for all values of before-hand and store them in a table.

### Author's response

The reviewer finally suggests that one could calculate f*log(f) for all values beforehand and store them in a table. However, this suggestion is not practical. Both S and H are real numbers. The number of all real numbers within the interval, e.g., [0,1] is infinite. Therefore, the table that contains 'all values' cannot be saved, unless with the infinitely large computer storage, which, however, does not exist.
